# Application of Intelligent Medical Sensing Technology

**DOI:** 10.3390/bios13080812

**Published:** 2023-08-13

**Authors:** Jie Fu, Qiya Gao, Shuang Li

**Affiliations:** Academy of Medical Engineering and Translational Medicine, Tianjin University, Tianjin 300072, China; fujie314159@tju.edu.cn (J.F.); 3019207445@tju.edu.cn (Q.G.)

**Keywords:** intelligent medical sensing technology, integrated circuits, health monitoring, physical sensor, chemical sensor, biosensor

## Abstract

With the popularization of intelligent sensing and the improvement of modern medical technology, intelligent medical sensing technology has emerged as the times require. This technology combines basic disciplines such as physics, mathematics, and materials with modern technologies such as semiconductors, integrated circuits, and artificial intelligence, and has become one of the most promising in the medical field. The core of intelligent medical sensor technology is to make existing medical sensors intelligent, portable, and wearable with full consideration of ergonomics and sensor power consumption issues in order to conform to the current trends in cloud medicine, personalized medicine, and health monitoring. With the development of automation and intelligence in measurement and control systems, it is required that sensors have high accuracy, reliability, and stability, as well as certain data processing capabilities, self-checking, self-calibration, and self-compensation, while traditional medical sensors cannot meet such requirements. In addition, to manufacture high-performance sensors, it is also difficult to improve the material process alone, and it is necessary to combine computer technology with sensor technology to make up for its performance shortcomings. Intelligent medical sensing technology combines medical sensors with microprocessors to produce powerful intelligent medical sensors. Based on the original sensor functions, intelligent medical sensors also have functions such as self-compensation, self-calibration, self-diagnosis, numerical processing, two-way communication, information storage, and digital output. This review focuses on the application of intelligent medical sensing technology in biomedical sensing detection from three aspects: physical sensor, chemical sensor, and biosensor.

## 1. Introduction

A sensor is a detection device that can convert measured data into electrical signals output according to certain rules in order to meet the requirements of information transmission, processing, storage, display, recording, and control [1,2,3]. The performance indicators of sensors usually include sensitivity, resolution, linearity, repeatability, stability, accuracy, and so on [4,5,6]. Sensitivity represents the change in the output of a sensor caused by a change in unit input. It is usually obtained by differentiating the output of the sensor characteristic curve from the input. The higher the sensitivity value, the more sensitive the sensor is. Resolution refers to the ratio between the minimum input increment that a sensor can detect and the full scale, which is used to indicate the minimum measurable input change of the sensor. Linearity represents the degree of deviation between the calibration curve of the sensor and the fitted straight line. The higher the linearity, the smaller the deviation, indicating better sensor performance. Repeatability characterizes the degree of non-coincidence of multiple characteristic curves measured by a sensor under the same working condition when the input is continuously changed multiple times in the same direction. The ideal repeatability error of the sensor should be 0, which means that the curves measured multiple times are completely coincident. Stability refers to the ability of a sensor system to maintain performance for a considerable period, usually expressed as the time difference between the system output and the initial calibration output after a specified time interval at room temperature. The three main indicators that affect stability are time zero drift, zero temperature drift, and sensitivity temperature drift. Accuracy characterizes the maximum deviation between the sensor’s indicated value and the measured true value. From the above indicators, it can be analyzed that all indicators of the sensor cannot be optimal at the same time. For example, sensitivity and stability are contradictory, while high sensitivity indicates low stability, and vice versa. The relationship between resolution and accuracy is that high resolution is a necessary condition for high accuracy, but not a sufficient condition. When selecting a sensor, accuracy should be the first consideration, followed by resolution. In practical applications, because the environment where the sensor located is usually complex, which may be confounding at the same time, and because the signal strength range of the target to be measured is not clear, the selectivity and linear detection range of the sensor must also be taken into account. The good selectivity of the sensor means that it has strong anti-interference ability during operation, and the wide linear detection range means that it can widely receive various signal strengths without causing an overflow.

The intelligent sensor is equipped with a microprocessor to collect, process, and exchange information [7,8,9]. It is the product of the combination of sensor integration and microprocessor. The main distinguishing feature of the intelligent sensor from the general sensor is that it has functions such as self-calibration self-correction, and automatic compensation, as well as the ability to store data and process information [10,11]. Intelligent sensors can correct various deterministic system errors through artificial intelligence, such as nonlinear errors in sensor input and output, server error, zero-point error, forward and backward stroke error, etc., and can also appropriately compensate for random errors and reduce noise, greatly improving sensor accuracy [12,13,14]. In addition, the miniaturization of intelligent sensor systems eliminates some unreliable factors of traditional structures, improves the anti-interference performance of the entire system, and has good stability. Finally, intelligent sensors can achieve comprehensive measurement of multiple sensors and parameters and can expand the measurement and usage range through programming, with certain adaptive capabilities. Its digital communication interface function can directly send data to terminals of various application systems for processing. Regardless of the type of sensor, the electrical signal output by their conversion elements is generally weak and accompanied by noise. Therefore, before signal acquisition, a signal conditioning circuit is required to amplify and filter the signal. The interface circuit further processes the electrical signal to produce an output consistent with the use of a measurement or control system [15,16,17]. In the end, the output electrical signal becomes a digital signal after analog-to-digital, and the control system resolves the state of the measured amount from the digital signal and views it through the communication or uses it directly for regulation.

In summary, the construction of intelligent sensors requires three elements: sensitive components, interface circuits, and communication interfaces. Since microprocessors are already mentioned in the definition of intelligent sensor sensors, they are not listed here as necessary elements. As the medium of signal type conversion, the selection of sensitive elements is particularly important, which directly affects the performance parameters of the whole sensor, such as signal-to-noise ratio, resolving power, accuracy, etc. Typically, the sensitive element is more dependent on the environment. Changes in temperature, humidity, light intensity, and other environmental factors may cause instability of the sensitive element [18,19]. The self-calibration function of intelligent sensors before working makes them more widely used than ordinary sensors. It is necessary to emphasize that the calibration of a sensor relies on a known calibration signal and the calibration process will inevitably generate errors that affect sensor accuracy [20]. Nevertheless, the sensor still needs to be calibrated after a certain period. Interface circuits are often defined as processing circuits for the output signals of sensitive components, which include impedance conversion circuit, amplifier circuit, current-voltage conversion circuit, bridge circuit, frequency-voltage conversion circuit, charge amplifier circuit, filter circuit, etc. The main functions of interface circuits are shown in Table 1. The amplifier circuit and filter circuit are the basic interface circuits for sensors. The current-to-voltage conversion circuit is mainly used in current-type sensors, and the impedance amplifier plays a conversion role in this circuit. The frequency-voltage conversion circuit builds a linear relationship between input frequency and output voltage.

The current trend in intelligent sensor design is to digitize the analog signal output from the sensor as soon as possible, and then conduct signal conditioning by software, such as filtering, linearization, cross-sensitivity compensation, etc., which reduces the difficulty of the sensor hardware circuit, enhances the flexibility of signal processing, and avoids signal attenuation and interference in circuit transmission. In practical applications, it is extremely common to replace some functions of hardware circuits with algorithms. This method can achieve rapid interconnection of sensors and intelligent control of systems, which is in line with the rapid development trend of integrated circuits and digital signal processing. Commonly used communication interfaces for intelligent sensors are serial peripheral interfaces, inter-integrated circuits, universal synchronous asynchronous receiver transmitters, etc. In addition to communication interfaces, intelligent sensors should also have high-speed data processing capabilities, visual control terminals, and excellent human-computer interaction interfaces [21,22]. Due to the above advantages, smartphones are widely used as terminals for intelligent sensors, effectively developing highly sensitive portable sensor detection systems.

**Scheme 1:**
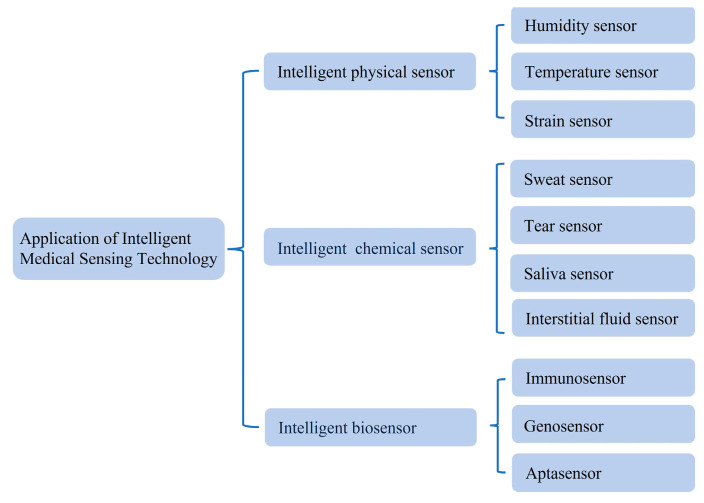
Application of intelligent medical sensing technology.

In this review, we first introduce the calibration part of an intelligent medical sensor and describe various calibration methods and their applicable scenarios in detail, which is the key to the normal operation of sensors (Scheme 1). Secondly, we discuss the applications of intelligent medical sensing technology in intelligent physical sensors, intelligent chemical sensors, intelligent biological sensors, and wearable sensors proposed in recent years. We divide intelligent physical sensors into humidity sensors, temperature sensors, and strain sensors based on the different detection objects. The introduction of intelligent chemical sensors focuses on the detection of human body fluids and is divided into four parts: sweat sensors, tear sensors, saliva sensors, and interstitial fluid sensors. According to the different biometric components in intelligent biosensors, they are divided into immunosensors, genosensors, and aptasensors. Finally, we discussed the prospects and challenges of the development of intelligent medical sensing technology and summarized solutions to its limitations.

## 2. Calibration

The process of experimentally determining, under predetermined measurement conditions, the correspondence between a sensor’s input and output is known as calibration. The sensor has to be recalibrated if any of its indicators have changed as a result of the external environment or if it has been some time since the last calibration. Usually, the sensor is calibrated once in the factory and then again according to user requirements. All sensors require calibration in order to function normally since it is the only way for the sensor to obtain calibration equations and because it establishes the accuracy of the sensor’s analog signal output.

### 2.1. Introduction to Calibration Methods

The basic method of calibration is to input a known signal to the sensor and obtain the output signal at the same time, thus obtaining a series of curves characterizing the correspondence between the two. In all calibration results, the linear relationship is undoubtedly the simplest and most conducive to sensor calibration. When the calibration result is non-linear, a reasonable data processing method is usually selected to linearize it. When calibrating sensors, the accuracy of the measuring equipment used is usually an order of magnitude higher than that of the sensor to be calibrated for error minimization calibration. Specifically for a piezoelectric pressure sensor, a piston manometer is used to generate a standard force of known magnitude on the sensor, which will output a corresponding charge signal, which is then measured by a standard detection device of known accuracy to obtain the magnitude of the charge signal, resulting in a set of input-output relationships. The relationship can generally be described by an equation whose parameters are referred to as calibration factors. Such a series of processes is the calibration process for piezoelectric pressure sensors.

For the purpose of ensuring measurement accuracy, self-calibrating sensors employ a variety of technical techniques to remove drift. In some ways, self-calibration is the same as recalibration prior to every measurement, which can remove the sensor system’s drift in temperature and time. This requires the integration of the corresponding calibration signal source as well as the calibration control circuit or algorithm in the sensing system. If the calibration signal source cannot be integrated into the system, it can only be obtained from an external source, which improves calibration accuracy but is less convenient. The calibration control circuit includes cross-sensitivity compensation, differential compensation, background calibration, etc. In summary, the self-calibration of the intelligent sensor is a process of automatically collaborating with the microcontroller and actuator to calibrate sensor components. Although self-calibration cannot completely take the place of an actual calibration, it may reduce the number of calibration points required for a given accuracy condition or extend the time between calibrations. The information storage capacity of intelligent sensors is the basis for successfully accessing the chain of calibration during self-calibration. The intelligent sensor saves calibration data in an electrically erasable programmable read-only memory (EEPROM) during calibration, which can be recalled by the microprocessor at any time when it is needed. EEPROM is generally a separate integrated chip that can be used “plug-and-play” on the same sensor, which means that the relevant calibration data does not need to be updated separately when replacing or recalibrating the same intelligent sensor.

### 2.2. Self-Calibration by Combining Multiple Sensors

**Figure 1:**
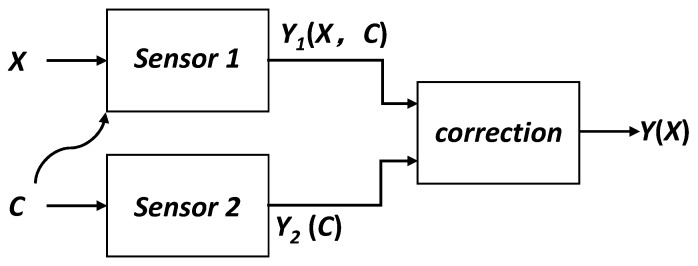
Cross-sensitivity compensation.

Figure 1 shows the principle of cross-sensitivity compensation, where both the interference signal C and the signal to be measured X can cause a change in the output signal of sensor 1. To eliminate the influence of the interference signal C, the cross-sensitivity is compensated for by the detection of the interference signal C by sensor 2. The effectiveness of this method depends on the reproducibility of the cross-sensitivity of sensor 1 to the interfering signal C [23]. If this cross-sensitivity is highly variable over time, then the improvements that can be obtained may be limited. Where sensors have a defined cross-sensitivity, the addition of sensors can significantly improve overall performance.

**Figure 2:**
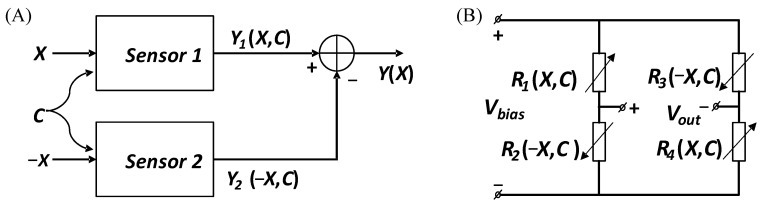
Differential compensation and application. (**A**) Differential compensation. (**B**) Wheatstone bridge.

Differential compensation in which two identical sensors are used to measure two signals of the same magnitude and opposite phase that are to be measured (Figure 2A). As the interfering signal C acts on both sensors simultaneously, the resulting errors are canceled out in the subsequent calculation. Figure 2B shows an example of a typical differential compensation circuit called the Wheatstone full-bridge [24]. R1 and R2 are shown as a set of strain gauges placed perpendicular to each other, as are R3 and R4. The changes caused by temperature on the four strain gauges should be the same and the absolute value of the change in resistance of the four strain gauges should be the same when the object is deformed. A simple calculation shows that the final output voltage Vout is proportional to the absolute value of the change in resistance [25]. There is no non-linear error and the sensitivity is numerically equal to the supply voltage Vbias.

**Figure 3:**
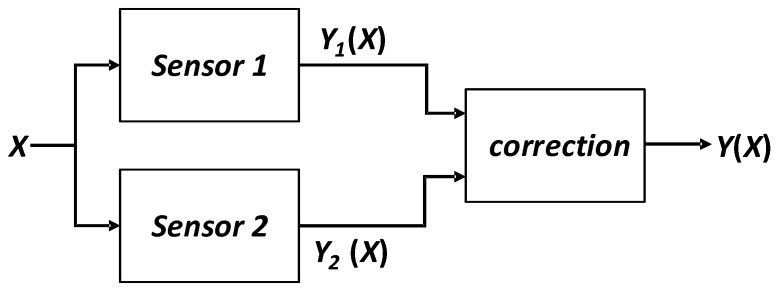
Background calibration.

Background calibration is the use of two different sensors to measure the same signal, as shown in Figure 3. The two sensors have different characteristics: for example, one of them is more accurate but has a slow response time, and the other one is less accurate but has a fast response time. In this case, the system often compares the data from the two sensors and corrects the output with a faster response. The combination of these two sensors produces a fast and accurate measurement system.

## 3. Intelligent Physical Sensor

Intelligent physical sensors convert the state of the object being measured into an electrical signal that can be recognized by certain physical effects. This paper focuses on the medical applications of humidity sensors, temperature sensors, and strain sensors when used as wearable sensors.

### 3.1. Humidity Sensor

**Figure 4:**
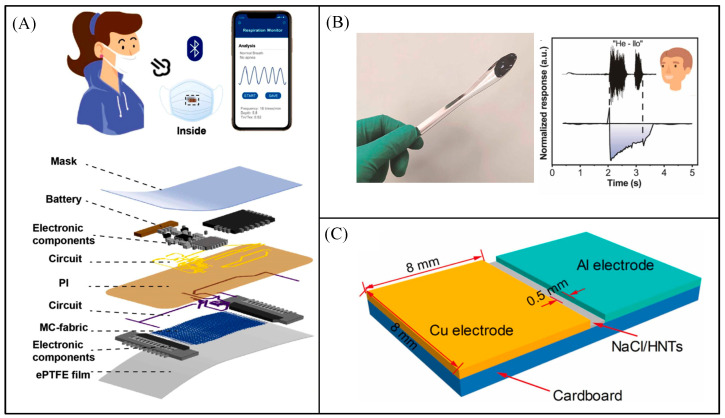
Humidity sensor. (**A**) Wireless data transmission and the exploded view of the detection tag when installed in the mask [28] Copyright 2022 Elsevier. (**B**) SA-MXene ink and Test curve of a humidity sensor based on SA-MXene [29] Copyright 2022 Elsevier. (**C**) NaCl/HNT humidity sensor [30] Copyright 2023 Elsevier.

One effective strategy for measuring respiratory humidity is to use ionic conductivity to characterize changes in humidity. MXene nanosheets can be used as sensing elements in humidity sensors due to their high conductivity, high surface area, and hydrophilicity [26,27]. The various functional end groups of MXene nanosheets (such as -OH, -O, and -F) make them highly sensitive to the adsorption of water molecules. With the adsorption of water molecules, the tunneling distance of MXene nanosheets increases, which ultimately leads to a decrease in ionic conductivity. Figure 4A shows the MXene-based humidity sensor being integrated into the breathing mask, which transmits data to smartphone via Bluetooth [28]. This humidity sensor can identify different breathing patterns, including normal breathing (12 to 20 breaths/min), rapid breathing (>24 breaths/min), slow deep breathing (<12 breaths/min), and apnea. Figure 4B depicts a humidity sensor based on SA-MXene composite material, which has enhanced resistance oxidation and sensitivity [29]. In addition, SA-MXene is made into ink and has been proven suitable for screen printing, which is extremely convenient for the manufacture of flexible sensors. Figure 4C shows a humidity sensor based on NaCl/HNT, which uses cardboard with high thickness and stability as the substrate to form a planar electrolytic cell structure [30]. The characterization and humidity sensing test results show that the sensor has a wide humidity sensing range and a high response.

### 3.2. Temperature Sensor

The sensitive element used in temperature sensors is the thermistor, which is a resistor whose resistivity changes significantly with temperature and is divided into PTC thermistors and NTC thermistors. NTC thermistors are widely used because of their advantages, such as high measurement accuracy, interchangeability, and reliability. CPCs are ideal for making flexible temperature sensors due to their flexibility, good processability, and light weight. However, manufacturing a flexible CPC-based temperature sensor with a linear NTC effect remains a challenge, as CPCs typically exhibit non-monotonicity between resistance and temperature. Most critically, flexible materials can cause temperature measurement errors when they undergo bending deformation.

**Figure 5:**
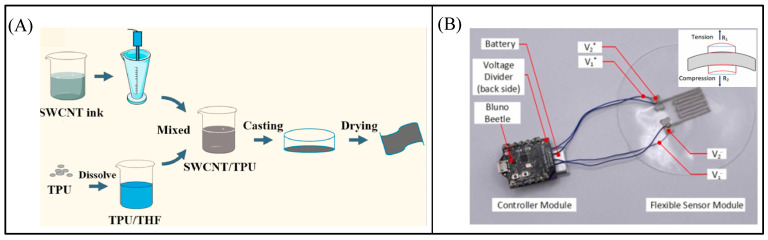
Temperature sensor. (**A**) TPU/SWCNTs composites [31] Copyright 2022 Elsevier. (**B**) Temperature sensor made by differential compensation principle [32] Copyright 2023 Elsevier.

Zhu et al. prepared TPU/SWCNTs composites that exhibit a monotonic and linear NTC effect over a temperature range of 30–100 °C (Figure 5A), which can be designed as highly flexible and sensitive temperature sensors. However, measurement errors due to the deformation of flexible materials cannot be avoided [31]. Figure 5B shows a sensing system that uses two identical resistive temperature sensors using the principle of differential compensation to compensate for changes in resistance due to deformation and measure temperature with minimal error [32]. When the sensor is bent on a curved surface, the resistance of R1 increases due to tension and the resistance of R2 decreases due to compression. The sum of resistance can reduce the effect of bending and the remaining errors can be minimized by calibration.

### 3.3. Strain Sensor

Most conventional strain sensors are based on metal and semiconductor materials, which are less portable, flexible, and wearable. The current mainstream strain sensors are based on flexible materials to construct flexible strain sensors, such as PDMS, PET, PI, PE, and PU. The development of dielectric materials with higher dielectric constants, lower leakage currents, higher dipole densities, higher current densities, higher energy densities, and fast discharges, as well as lower losses, and thus the preparation of these materials as dielectric layers for flexible electronic devices has become a hot research topic. Flexible strain sensors are widely used in electronic skins, wearable electronic devices, and soft robotics as flexible, stretchable electronic devices that efficiently convert deformations caused by external forces into electrical signals for motion detection and health monitoring purposes.

**Figure 6:**
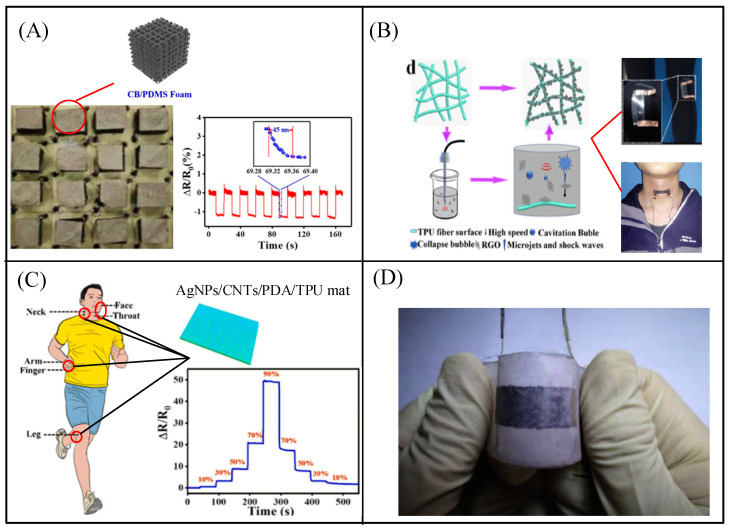
Strain sensors. (**A**) CB/PDMS−based flexible strain sensor [33] Copyright 2019 Elsevier. (**B**) TPU/RGO based strain sensor [36] Copyright 2018 Elsevier. (**C**) AgNPs/CNTs/PDA/TPU−based strain sensor [37] Copyright 2023 Elsevier. (**D**) GF/NF strain sensors being bent and twisted [38] Copyright 2019 Elsevier.

As shown in Figure 6A, Zhai et al. reported a flexible sensor based on a CB/PDMS foam sensor with a high durability of over 15,000 cycles [33]. Chen et al. encapsulated the sensor with a PMMA layer approximately 5 µm thick to improve durability [34]. Kweon et al. reported a flexible strain sensor based on a PVDF copolymer that operated stably over 10,000 strain cycles [35]. Wang et al. reported a flexible strain sensor consisting of a TPU fiber pad and RGO (Figure 6B), which exhibits good durability due to the strong bond between the TPU fibers and the RGO [36]. Figure 6C shows a tensile strain sensor based on AgNPs/CNTs/PDA/TPU pad composites [37]. Using a TPU fiber pad as a stretchable substrate, PDA is anchored to the surface of the TPU, improving the interface between the substrate and nanofillers. Finally, CNT and AgNPs are modified on the surface of the PDA/TPU, and the introduction of PDA effectively prevents the oxidation of AgNPs. Figure 6D shows a wearable strain sensor based on GF/NF. The flexible and high-conductivity graphene films prepared by the thermal expansion compression molding process have good electrical properties and stability [38]. In addition, a GF/NF strain sensor was used to detect multiple joints in the human body. The data results show that the GF/NF strain sensor has high sensitivity and good repeatability. The strain sensor has a wide detection range, high sensitivity, stable response, and long-term durability, and can be used for human motion detection. The sensitivity of flexible sensors varies depending on their active material, structure, and sensing mechanism. The ideal flexible strain sensor should have high sensitivity, wide strain sensing range, satisfactory repeatability, sample preparation process, and versatility, allowing the sensor to monitor the human body faster, easier, more accurately, and more comprehensively.

## 4. Intelligent Chemical Sensor

Physical sensors only provide the electrophysiological information of the human body, and for further monitoring of body fluids and metabolites that contain disease markers, chemical biosensors are needed. As the need for personal health monitoring continues to grow, it has been necessary to develop a sensor that can provide diverse and dynamic detection of analytes [39]. Wearable sensors are an increasingly attractive solution because of the advantages mentioned above, which integrated electronic sensors for non-invasive measurements of biomarkers [40,41]. These sensors can collect and analyze biological fluids, including sweat, tears, saliva, and interstitial fluid. Promoted by the material innovation from traditional metal and semiconductor materials to flexible/stretchable 2D material, polymer, and biomaterials, wearable chemical sensors have shown great prospects in healthcare and environmental monitoring applications by interfacing with different signal transmission technologies, such as Bluetooth, Wi-Fi, and RF.

### 4.1. Sweat Sensor

**Figure 7:**
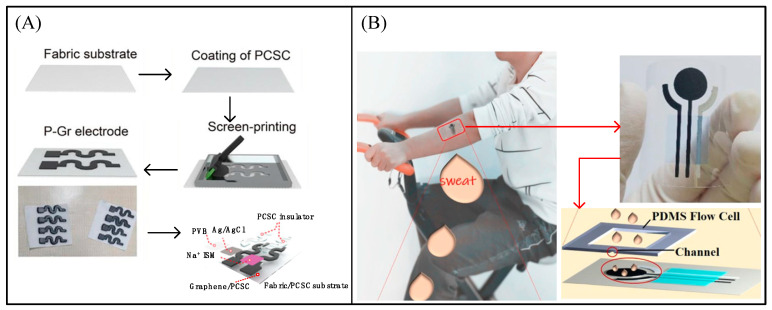
Wearable sweat sensors. (**A**) Fabrication of P-Gr electrode by screen-printing [44] Copyright 2023 Elsevier. (**B**) MIP formation and biosensing [46] Copyright 2020 Elsevier.

Sweat is particularly popular because of the easy acquisition of it from the large skin surface, and it contains abundant markers such as metabolites, proteins, and hormones [42]. Wearable sweat sensors combine the benefits of non-invasive sweat sampling and wearable real-time measurement to provide a powerful platform for monitoring the rich biochemical composition of sweat for physiological conditions [43]. Figure 7A shows a self-repairing, robust, and electrically conductive ink by combining Gr with PCSC. The results showed that the resulting P-Gr conductor had a high conductivity of 1243 Sm^−1^ and maintained a tensile strain of 213%. The electrode printed with this ink has almost no resistance change under 200% tensile strain [44]. The electrode is used to make a sweat sensor to detect sodium ions and by integrating the sensor with a wireless electronic module. Jia et al. prepared a skin tattoo biosensor consisting of conductive inks directly attached to the wearer’s skin for lactate sensing [45]. The sensor has good selectivity for lactic acid and still shows a good linear response at lactic acid concentrations up to 20 mM, enabling continuous monitoring of lactic acid content in human sweat. Figure 7B shows a flexible chemical biosensor based on AgNWs and MIP for monitoring lactic acid in sweat. The lactate sensor was applied to the epidermis of volunteers to monitor lactic acid in vivo based on differential pulse voltammetry measurements [46]. The results showed that the calibration curve had good linearity (correlation coefficient of 0.995) and specificity within the set concentration range, and the LOD of lactic acid detection in PBS was estimated to be as low as 0.22 μM (S/N = 3).

### 4.2. Tear Sensor

**Figure 8:**
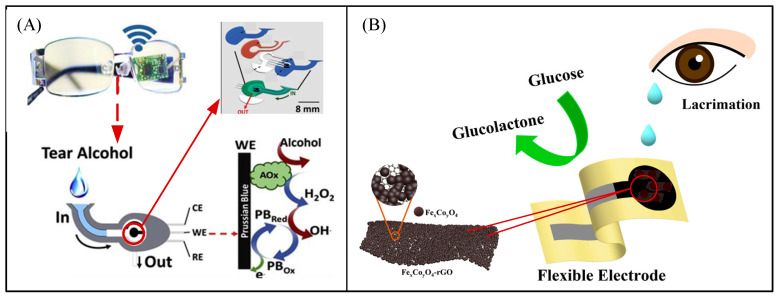
Wearable tear sensors. (**A**) Alcohol detection integrated into glasses [49] Copyright 2016 Elsevier. (**B**) Glucose detection [50] Copyright 2019 Elsevier.

Tears contain a variety of biomarkers, and as a more readily available biological fluid than blood, the concentrations of metabolites in tears reflect their levels in blood [47]. Wearable tear-based sensing systems have been reported to detect a wide range of analytes, including glucose and lactate [48]. Most of these systems integrate chemical sensors into an eyewear-based platform that has integrated electronics and is commonly used for daily monitoring. Sempionatto et al. mounted a line fluid device on the nose bridge pad of an eyeglass, thereby enabling a non-invasive wearable tear biosensor system. This system requires stimulation of the eye before tears can be collected for real-time monitoring of different target analytes, such as alcohol, vitamins, and glucose in tears (Figure 8A) [49]. Figure 8B shows that a chemical sensor for analyzing glucose in tears, where a suspension consisting of Fe, Co bimetallic oxides, and RGO was added dropwise to the electrode surface, and Nafion solution was added dropwise after the suspension dried to prevent the material from falling off [50]. This non-enzymatic glucose detection platform is characterized by high sensitivity, high selectivity, and low detection limits, and can be used for dynamic analysis of glucose content in tear fluid.

### 4.3. Saliva Sensor

Saliva is a biofluid that is more readily available non-invasively than sweat or tears, and it has an abundance of components, such as electrolytes, metabolites, and protein biomarkers that provide a timely picture of the oral cavity [51]. However, saliva sensors have significant limitations in terms of where they can be worn compared to sweat sensors. In addition, it is challenging to fix the sensor on the teeth and effectively prevent sensor wear.

**Figure 9:**
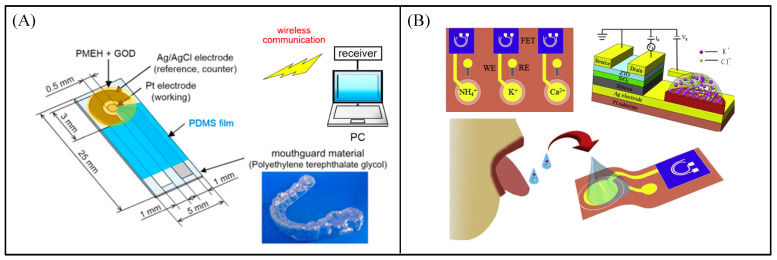
Wearable saliva sensors. (**A**) Glucose monitoring [52] Copyright 2016 Elsevier. (**B**) Flexible ISFET of detecting ions [54] Copyright 2019 Elsevier.

Figure 9A shows a saliva sensor used to detect glucose, which can achieve long-term real-time non-invasive monitoring through wireless communication modules. The sensor combines Pt and Ag/AgCl electrodes onto a tooth guard bracket with an enzyme film. The electrode is formed on the PETG surface of the tooth guard [52]. The Pt working electrode is coated with a GOD film. The results show that the glucose sensor can perform high sensitivity detection in the glucose range of 5–1000 µ mol/L, which covers the range of glucose concentrations found in human saliva. Wang’s group reported a tooth-care lactate biosensor based on a three-electrode system, with the specific scheme of modifying lactate oxidase on the sensor to catalyze lactate in saliva. The high repeatability and selectivity of the sensor create favorable conditions for continuous monitoring of the oral cavity with a Bluetooth transceiver for communication [53]. Bao et al. used a 3D printing method to directly connect an FET and an ISE to produce a flexible ISFET (Figure 9B). Then, the sensitivity and selectivity of mixed ISFETs were further studied. Finally, the possibility of ISFET detecting NH^4+^, K^+^, and Ca^2+^ in artificial saliva from interfering ion mixtures was demonstrated, expanding the application of ISFET as the next-generation electronic tongue in the field of non-invasive real-time health monitoring [54]. M. S. Mannoor et al. reported a wireless graphene bacterial sensor mounted on tooth enamel. The self-assembled antimicrobial peptide is immobilized on graphene as an artificial receptor, and the conductivity of the graphene film changes when the immobilized peptide binds to a specific bacterial target. Finally, the signal is read by an RF reading device [55]. Tseng et al. reported an RF-Trilayer saliva sensor [56], which consists of an active layer sandwiched between two reverse-facing split ring resonators where the dielectric sensing signal is wirelessly coupled to the reader to achieve food consumption analysis, such as pH, salinity, biomolecules, and alcohol.

### 4.4. Interstitial Fluid Sensor

Interstitial fluid exists between cells and capillaries produced by the filtration of blood plasma through capillaries, and its composition is similar to that of blood plasma. Interstitial fluid lacks some of the proteins present in blood plasma, which is because some of the large protein components cannot pass through when blood plasma passes through capillaries for fluid exchange [57]. Because of the close association of interstitial fluid with plasma, the detection of biomarkers, metabolites, and drugs in interstitial fluid is essential [58,59,60,61]. To minimize damage to the patient’s skin, techniques such as microneedle [62] and electroporation [63] have been developed to obtain interstitial fluid. However, these extraction techniques are not yet suitable for clinical applications due to different equipment and methods, long extraction times, and generally low extraction volumes [64]. The development of wearable devices based on the biochemical detection of interstitial fluid by combining chemical sensors with microneedles or patches [65,66] for extracting interstitial fluid is still the focus in the field of health monitoring.

**Figure 10:**
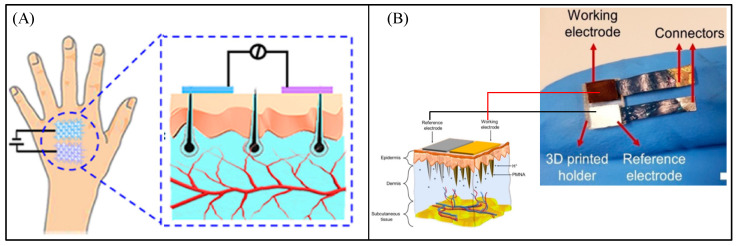
Wearable interstitial fluid sensors. (**A**) Reverse iontophoresis-based glucose detection [67] Copyright 2021 Elsevier. (**B**) PMNA-based sensing patch for pH detection [68] Copyright 2023 Elsevier.

Yao et al. proposed a noninvasive glucose sensor that can extract interstitial fluid and perform sensitive detection of glucose by the current applied between electrodes with a graphene/carbon nanotube/glucose oxidase composite textile as the working electrode (Figure 10A) [67]. The sensor was verified to be capable of continuous noninvasive detection by comparison with the detection results of a glucose meter. Figure 10B shows that Dervisevic et al. developed a high-density PMNA-based sensing patch that uses microneedle technology to monitor the skin pH [68]. The sensing patch was tested to detect pH 4.00–8.60 with a sensitivity of 62.9 mV/pH and an accuracy of ±0.036 pH units.

## 5. Intelligent Biosensor

Biosensing technology is a high technology that grows from the interpenetration of various disciplines, such as biology, chemistry, physics, medicine, and electronics. A biosensor is an analytical system consisting of immobilized sensitive materials, appropriate physicochemical transducers (such as oxygen electrodes, photosensitive tubes, field-effect tubes, piezoelectric crystals, etc.), and signal amplification devices [69]. Due to its good selectivity, its high sensitivity, its fast analysis, its low cost, the possibility of online continuous monitoring in complex systems, and especially its high degree of automation, miniaturization, and integration [70], the biosensor has vigorous and rapid developed in recent decades. In 1962, Clark Jr produced the first generation of glucose biosensors by containing glucose oxidase in a polyacrylamide colloid and immobilizing this colloidal film on the tip of a septum oxygen electrode. In clinical medicine, biosensors using enzymes as sensitive materials have been successfully applied to the detection of blood glucose, lactate, vitamin C, uric acid, urea, glutamate, transaminases [71], etc. In addition, immunosensors, genosensors, and aptasensors are all biosensors with their characteristics and applications.

### 5.1. Immunosensor

**Figure 11:**
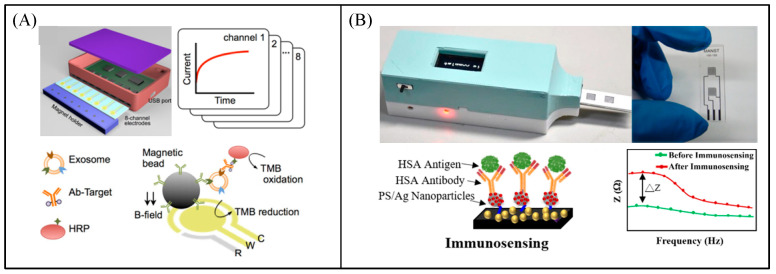
Immunosensors. (**A**) Integrated exosome biosensor [73] Copyright 2016 ACS Nano. (**B**) Microalbuminuria monitoring by impedance reading and wireless transmission for cloud computing [74]. Copyright 2019 Elsevier.

Owing to their high affinity and specificity, antibodies are one of the most effective detection elements used in biosensors [72], and biosensors utilizing antibodies are referred to as immunosensors. Lee’s group presented an integrated assay with eight channels for rapid EV screening (Figure 11A). Magnetic beads captured and labeled EVs and then assessed them via immunoassay [73]. The device simplifies vesicle isolation and has demonstrated rapid extracellular vesicle profiling in clinical samples. Shaikh et al. fabricated a simple disposable immunosensor for the point-of-care testing of microalbuminuria [74]. The immunosensor was performed on the surface of carbon IMEs printed on flexible PET sheets (Figure 11B).

### 5.2. Genosensor

**Figure 12:**
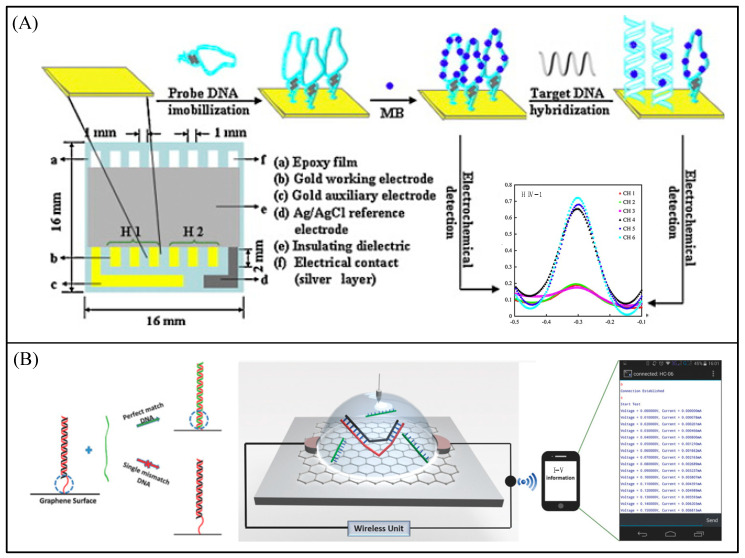
Genosensors. (**A**) Label-free DNA biosensor array [76] Copyright 2010 Elsevier. (**B**) Wireless label-free graphene−based FET genotyping platform [77]. Copyright 2018 Advanced Materials.

Genosensors are a widely reported class of biosensors [75]. The application in clinical disease diagnosis is the greatest advantage of genosensors, which can help physicians understand disease processes at the level of DNA, RNA, proteins, and their interactions. Zhang et al. developed a label-free DNA biosensor array [76] consisting of six gold working electrodes, a gold auxiliary electrode, and an Ag/AgCl reference electrode (Figure 12A). The six gold working electrodes were equally divided into two groups, each with a kind of thiolated hairpin-DNA probe modified on the surface to simultaneously detect Human Immunodeficiency Virus (HIV) oligonucleotide sequences HIV-1 and HIV-2. The biosensor array showed good specificity without the obvious cross-interference. Hwang et al. developed a Wireless label-free graphene-based FET genotyping platform [77], which was able to discriminate single nucleotide polymorphism with high sensitivity based on the immobilization of DNA nanotweezers onto the graphene surface (Figure 12B). DNA strand displacement in the presence of the target DNA and the subsequent DNA nanotweezers opening causes the switching of different involved strands, ultimately translated as a charge difference before and after. Guedes has reported a genosensor for detecting Late-Life Depression. The basic principle is that a gold electrode is modified with a DEP1S probe (Au/DEP1S), and, subsequently, a plasma sample containing miR-184 is added (Au/DEP1S:miR-184). After the hybridization process, ethidium bromide is added (Au/DEP1S:miR-184/EB) and its oxidation peak is monitored by differential pulse voltammetry. The results showed that the genosensor presented a linear response, detecting from 10^−9^ M to 10^−17^ M of miR-184 in plasma (1:10, *V*/*V*) [78]. In addition, the biosensor did not significantly lose its current response after 10 repetitions, and it still had a response of 72% after 50 days of storage, directly proving its excellent repeatability and stability.

López-Mujica modified MWCNTs Av bDNAp and MWCNTs Av bDNAp_1_ onto electrodes to develop two genosensors for quantitative detection of SARS-CoV-2 nuclear acid with response signals of impedance and current, respectively [79]. When the concentration of the target detection substance was in the range of 10^−18^ M to 10^−11^ M, the impedimetric genosensor had an excellent linear response (r^2^ = 0.994) and a sensitivity of (5.8 ± 0.6) × 10^2^ Ω M^−1^. An amperometric genosensor shows a fast and well-defined response, with a linear relationship between 10^−20^ M and 10^−17^ M and a sensitivity of (2.4 ± 0.3) × 10^2^ nA M^−1^ ((8 ± 1) × 10^3^ nA M^−1^ cm^−2^) (r^2^ = 0.99).

### 5.3. Aptasensor

**Figure 13:**
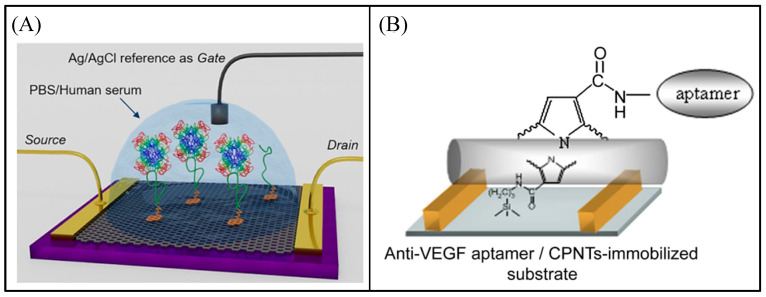
Aptasensors. (**A**) RGO based 2DBioFETs modified with PfLDH-specific aptamer [82] Copyright 2022 Elsevier. (**B**) FET sensor platform for VEGF detection [83]. Copyright 2010 Elsevier.

Among the wide variety of biomolecular recognition elements, aptamers are widely used due to their remarkable affinity, specificity, and ease of synthesis [80]. Aptamer is a specific structure formed by folding a single-stranded RNA or DNA molecule that binds to a specific target molecule [81]. Aptamer together with biosensing components provide a specific, selective, sensitive, easy-to-operate, fast, stable, and compact analytical platform called the aptasensor. Figueroa-Miranda et al. presented system-integrated RGO-based 2DBioFETs modified with PfLDH-specific aptamer as an optimal PoC solution for quantitative screening of malarial parasitemia (Figure 13A) [82]. Kwon et al. demonstrated the high-performance FET sensor, which could detect 400 fM of VEGF concentration, based on anti-VEGF RNA aptamer conjugated CPNTs (Figure 13B) [83]. Moreover, the CPNTs-aptamer FET sensors can be repeatedly used for VEGFs detection through the washing and rinsing processes. Representative sensor’s characteristic data in this article are shown in Table 2.

## 6. Conclusions and Perspective

An overview of intelligent sensing technology and its medical applications is given in this study. Intelligent medical sensing detection is a significant area of sensing technology as it is a comprehensive science and technology. Ergonomics, the uniqueness and complexity of biosignals, and the biocompatibility, dependability, and safety of intelligent medical sensors must all be fully considered in its design and implementation. From the perspective of the sensor manufacturing process, with the development of intelligent medical sensor technology, multiple types of sensors based on biomolecules, chemical organic/inorganic molecules, metal nanomaterials, carbon nanomaterials, quantum dots, nanocomposites, and polymers have been widely used in medical and health fields, while wearable sensors have become particularly prominent because of their light weight, flexibility, and excellent mechanical properties. Since the advent of nanomaterials, wearable sensors have achieved improved output characteristics through their high specific surface area, excellent electrocatalytic activity, good electron transfer ability, and conductivity. In addition, the abundance of sensor recognition components has significantly improved their sensitivity, specificity, detection ability, fast online analysis, and microanalysis. Importantly, they can monitor biomarkers in various biological fluids in a real-time, non-invasive, and continuous manner, which has become the main goal of wearable sensor development. Intelligent medical sensing technology has been able to integrate separation, enrichment, and measurement: it can selectively enrich through ion exchange reactions and surface coordination reactions of interface-modified materials, and improve the electron transfer ability between electroactive substances and electrode interfaces through modified materials. It can improve sensitivity by utilizing catalytic properties and selectivity by combining with biomolecular specific responses. From the perspective of sensor systems, the combination of wearable sensors and smartphones, with the help of low-power technology and self-powered technology, it is not difficult to achieve an intelligent wearable sensing system that can run for a long time. Combined with artificial intelligence and cloud medicine, advances in intelligent medical sensing technology bode well for the integration of wearable health systems. Real-time, continuous monitoring capabilities of wearable sensors provide a high-quality source of information for big data analysis, and successful integration will provide the means for patient-centered chronic disease management, reduce the frequency of clinical visits, and provide personalized, on-demand interventions.

In the latest non-invasive wearable sensor designs, the sensors should be able to detect multiple biomarkers through self-calibration to improve the reliability of wearable sensing as well as the ease of calibration. In wearable biosensors, biological recognition elements (including enzymes, antibiotics, antibiotics, peptides, DNA, aptamers, or living cells) are used, which improves the sensitivity and specificity of the sensor but also leads to increased production costs, complex production methods, and unsatisfactory stability and repeatability results. The emergence of new materials and detection principles effectively addresses the above shortcomings, such as nanoenzyme and MIP. Another challenge with wearable sensors is that their performance is limited by human physical activities, resulting in unstable connections (such as electrode displacement) or impedance changes, which directly lead to detection errors. This can process such artifacts by using multimodal sensor fusion, Kalman filter, short-term fast Fourier transform, wavelet transform, and other frequency domain algorithms to filter the signal to reduce errors.

## Figures and Tables

**Table 1:** Interface circuits.

Circuit	Function
Impedance conversion circuit	When the output is high impedance, it is converted to low impedance
Amplifier circuit	Amplify weak output signal
Current-voltage conversion circuit	Current output converted to a voltage
Bridge circuit	Change of resistance, capacitance, and inductance into current or voltage
Frequency-voltage conversion circuit	Frequency output converted to current or voltage
Charge amplifier circuit	Charge generated by the output of the electric field sensor is converted into voltage
Filter circuit	Noise of the sensor is eliminated by low-pass and band-pass filter

**Table 2:** Sensors characteristic data.

Analyte	Sensing Strategies	LOD	Sensitivity	Detection Range	Ref
Humidity	MXene/MWCNT electronic fabric	\	\	10–90% RH	[13]
Humidity	SA-MXene	\	\	11–97% RH	[14]
Temperature	TPU/SWCNTs	\	\	30–100 °C	[16]
Temperature	RTD	\	5.9 × 10^−3^/°C	20–40 °C	[17]
Strain	CB/PDMS	\	−1.12	0–91%	[18]
Strain	ACTM	0.1%	7030 & 4.8 × 10^5^ & 2 × 10^6^	0–230% & 230–520% & 520–640%	[22]
Na+	PCSC/Gr/Na-selective membranes	10^−5.4^ M	−62.35 mV/log[Na]	10^−4^–10^−1^ M	[29]
Lactate	MIP-AgNWs	0.22 μM	4.5 μA/M	10^−6^–10^−1^ M	[31]
Glucose	GOD/POD	1.84 mM	1.4 nm/mM	0–20 mM	[32]
Glucose	FexCoyO4-RGO	0.07 μM	1510 μM cm^−2^ mA^−1^	0.1–906.4 μM	[35]
Glucose	G/CNTs/GOx	\	574.26 μA mM^−1^ cm^−2^ & 12.11 μA mM^−1^ cm^−2^	0–0.1 mM & 1.0–30 mM	[52]
miR-184	Au/DEP1S	10 automobiles L^−1^	0.15305 μA/log[miR-184]	10^−17^–10^−9^ M	[64]
PfLDH	Aptamer/BSA	0.78 fM	1.3/decade	0.78 fM–100 nM	[68]

## Data Availability

Not applicable.

## Abbreviations

SA-MXene: sodium ascorbate-decorated MXene; HNT: halloysite nanotube; PTC: positive temperature coefficient; NTC: negative temperature coefficient; CPCs: Conductive polymer composites; TPU/SWCNTs: flexible thermoplastic polyurethane/single-walled carbon nanotubes; PDMS: polydimethylsiloxane; PET: polyethylene terephthalate; PI: polyimide; PE: poly-ethylene; PU: polyurethane; CB/PDMS: carbon black/Polydimethylsiloxane; PMMA: polymethyl methacrylate; PVDF: polyvinylidene fluoride; RGO: reduced graphene oxide; GF/NF: nylon fabric encapsulated graphene film; Gr: graphene; PCSC: poly(1,4-cyclohexanedimethanol succinate-co-citrate); P-Gr: PCSC/Gr; AgNWs: Ag nanowires; MIP: molecular imprinting; PETG: polyethylene terephthalate; GOD: glucose oxidase; FET: field effect transistor; ISE: ion selective electrode; ISFET: ion selective field effect transistor; IMEs: interdigitated microelectrodes; CPNTs: carboxylated polypyrrole nanotubes; VEGFs: various concentrations of the target molecule.
